# Fine mapping of the major anthracnose resistance QTL *AnR*_*GO*_*5* in *Capsicum chinense* ‘PBC932’

**DOI:** 10.1186/s12870-019-2115-1

**Published:** 2020-05-01

**Authors:** Yuanyuan Zhao, Yiwei Liu, Zhenghai Zhang, Yacong Cao, Hailong Yu, Wenwen Ma, Baoxi Zhang, Risheng Wang, Jie Gao, Lihao Wang

**Affiliations:** 1 Key Laboratory of Vegetable Genetics and Physiology of the China Ministry of Agriculture, Institute of Vegetables and Flowers, Chinese Academy of Agricultural Sciences, No 12 Zhongguancun South Street, Beijing, 100081 People’s Republic of China; 2 College of Forestry and Horticulture, Xinjiang Agricultural University, 467 Xinjiang, Urumqi, 830052 People’s Republic of China; 3 Vegetable Research Institute, Guangxi Academy of Agricultural Sciences, No 174, East University Road, Nanning, Guangxi 530007 People’s Republic of China

**Keywords:** Pepper, Anthracnose, Fine mapping, *Colletotrichum scovillei*, QTL

## Abstract

**Background:**

*Colletotrichum* species are the causal agents of anthracnose, a major disease affecting the yield and quality of pepper (*Capsicum* spp.). *Colletotrichum scovillei* is widespread in China, has strong pathogenicity and drug resistance, and causes anthracnose disease in pepper fruits that severely reduces production. Previously, an anti-anthracnose locus *AnR*_*GO*_*5* was mapped to the P5 chromosome on the basis of analyses of fruit at the green mature stage. The aim of this study was to narrow down the interval of this locus and identify the gene responsible for conferring resistance.

**Results:**

On the basis of results of re-sequencing of *Capsicum chinense* ‘PBC932’ and *C. annuum* ‘77013’, we developed Kompetitive allele-specific PCR (KASPar) markers and insertion–deletion (InDel) markers linked to *AnR*_*GO*_*5* at the green mature fruit stage and used them to construct a genetic linkage map (42 markers, 24.4 cM in length). Using data obtained in phenotypic and genotypic analyses of BC_4_S_1_, BC_4_S_2_, and BC_4_S_3_ populations, *AnR*_*GO*_*5* was located between the markers P5in-2266-404 and P5in-2268-978 within a physical distance of 164 kb. This region contained five genes, including *CA05g17730*. *CA05g17730* encodes ‘R1C-3-like’ putative late blight resistance protein homologs. The transcript level of *CA05g17730* differed between ‘PBC932’ and ‘77013’. The structure of the *CA05g17730* gene also differed between ‘PBC932’ and ‘77013’.

**Conclusions:**

We narrowed down the QTL interval to a region containing five genes. These results will be useful for further research on the mechanisms of resistance to anthracnose, and for marker assisted selection for anthracnose-resistant capsicum lines.

## Background

Anthracnose disease is one of the major economic constraints to pepper (*Capsicum* spp.) production worldwide, especially in tropical and subtropical regions [1]. In wet seasons, the fruit yield losses caused by anthracnose at both the pre-harvest and post-harvest stages can be more than 80% [2]. Symptoms of anthracnose include sunken necrotic tissues and the presence of acervuli [3]. To date, anthracnose in pepper has been attributed to 24 *Colletotrichum* species [4]. Three species occur on pepper in China, .originally identified as *C. capsici*, *C. gloeosporioides* and *C. acutatum* [1, 3, 5], were recently reclassified as *C. truncatum* [6, 7], *C. siamense* and *C. scovillei* [8–10]. The pathogen *C scovillei* used be called *C. acutatum* was be selected for this study.

Anthracnose disease is usually controlled by applying fungicides, but these compounds can have negative effects on the environment and human health. Agricultural control to remove diseased fruit and clear drainage ditches is very labor intensive. Biocontrol agents such as *Bacillus* sp*.* and its putative catalase may be useful to protect pepper from anthracnose [11]. However, the development of resistant cultivars is the best long-term strategy to control the disease, and so it is an important goal for breeders. There is still little information available about the interactions between the host and the causal pathogens of pepper anthracnose [12]. Breeding for anthracnose resistance has been attempted in Asia for more than 20 years with little success [13].

The anthracnose-resistant cultivars *Capsicum chinense* ‘PBC932’ and *Capsicum baccatum* ‘PBC80’ were identified by the AVRDC-World Vegetable Center in 1998, and since then, they have been shared among Asian pepepr breeders [14]. They are the two major sources of anthracnose resistance in pepper crops in Thailand [15]. Differential host reactions that occurred in the different pepper genotypes was a result of specific host and pathogen interactions of different stages of fruit maturity. From samples of diseased pepper from 29 provinces of China, fifteen *Colletotrichum* species were identified, *C. fioriniae*, *C. fructicola*, *C. gloeosporioides*, *C. scovillei*, and *C. truncatum* being prevalent. *C. scovillei* was one of dominant species on pepepr in China and it is mostly restricted to East Asia [8, 9].

In a previous study on resistance to *C. capsici* (now *C. truncatum*), analyses of the frequency distribution of disease scores in an intraspecific *C. baccatum* ‘PBC1422’ × *C. baccatum* ‘PBC80’ cross suggested that a single recessive gene is responsible for resistance at the mature green fruit stage and a single dominant gene is responsible for resistance at the ripe fruit stage [16]. Quantitative trait locus (QTL) mapping of resistance to *C. scovillei* (former *acutatum*) and *C.truncatum* (former *capsici*) has been conducted using three resistance assessment methods: disease incidence, true lesion diameter, and overall lesion diameter. The major QTLs for resistance derived from *C. baccatum* ‘PBC81’ to *C. scovillei* (former *acutatum*) and *C. truncatum* (former *capsici*) were in different positions [17]. Resistance to *C. capsici* (now *truncatum*) in ‘PBC932’ was found to be controlled by a single recessive gene [15]. The ‘AR’ line is an anthracnose-resistant breeding line derived from *C. chinense* ‘PBC 932’. The resistance to *C .truncatum* (former *capsici*) derived from ‘AR’ was also found to be controlled by a single recessive gene [18].

Resistance to *C. acutatum* (now *scovillei*) derived from *C. chinense* ‘PBC932’ was reported to be controlled by two complementary dominant genes in green fruit, but by two recessive genes in red fruit [19]. The two dominant genes influencing *C. acutatum* (now *scovillei*) resistance were identified in an intraspecific *C. annuum* cv. ‘Bangchang’ × *C. chinense* ‘PBC932’ population [20]. A map containing 12 linkage groups (LGs) with 214 single nucleotide polymorphisms (SNPs) and 824 cM coverage was constructed from a pepper population derived from *C. annuum* ‘Bangchang’ × *C. chinense* ‘PBC932’, and two QTLs corresponding to anthracnose resistance in mature green fruit were identified on LG2 between two SNPs within an interval of 14 cM [14]. In another linkage map with 14 LGs, 385 markers (simple sequence repeat, SSR; insertion-deletion, InDel; and cleaved amplified polymorphic sequence, CAPS), and a length of 1310.2 cM, a main effect QTL and four minor effect QTLs for anthracnose resistance at the green mature stage were localized on the P5 chromosome [21].

Among many types of molecular markers, single nucleotide polymorphisms (SNPs) are attractive for breeding [22]. Kompetitive allele-specific PCR (KASPar) markers, which are designed from SNP loci, offer cost-effective and scalable flexibility for applications such as marker assisted selection and QTL fine mapping [23]. InDel polymorphisms (base insertions or deletions) are relatively abundant in the genome, and InDel markers have been developed and used in previous studies [24, 25]. In this study, we used KASPar and InDel markers to construct a map to identify QTLs for anthracnose resistance in a population derived from *C. annuum* ‘77013’ × *C. chinense* ‘PBC932’. Fine-mapping analyses allowed us to narrow down the interval of a QTL conferring resistance.

## Results

### Phenotypic identification

**Fig. 1:**
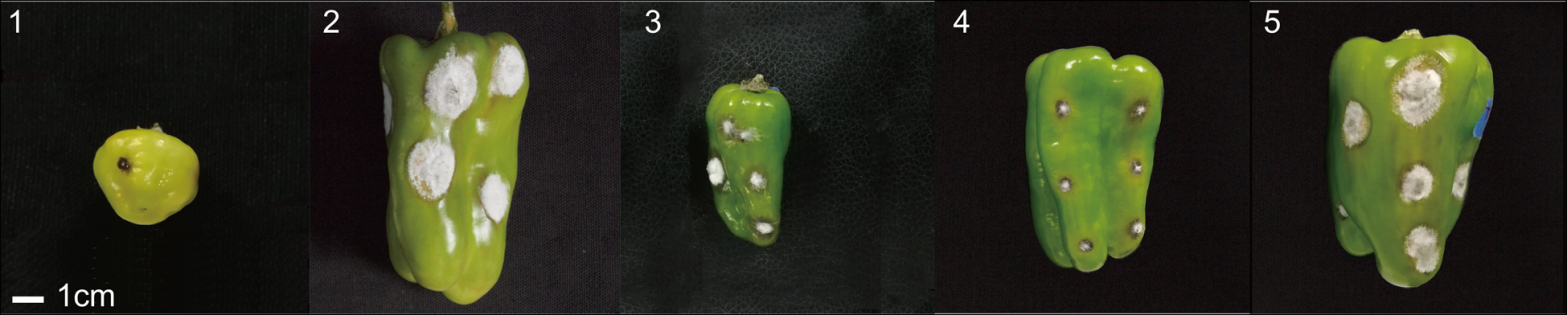
Disease symptoms on green fruit of ‘PBC932’ (1), ‘77013’ (2), F1 (77,013 × PBC932) (3), disease-resistant plants in BC_4_S_1_ population (4), and disease-susceptible plants in BC_4_S_1_ generation (5). 3.2 Fine positioning of *AnR*_*GO*_*5*

The disease resistance phenotypes were identified based on the following criteria: disease resistant (R): 0 mm ≤ O ≤ 12 mm; disease susceptible (S): O > 12 mm. All plants of ‘PBC932’ and F_1_ were R, and all plants of ‘77013’ were S. Inheritance of R was dominant. The BC_4_S_2_–1 and BC_4_S_2_–2 populations were derived from different disease-resistant individual plants of BC_4_S_1_. The BC_4_S_1_, BC_4_S_2_–1, and BC_4_S_2_–2 populations were normally distributed with respect to the distribution of R and S plants (Fig. 1). The separation ratios were 250:182, 140:102, 117:45, which fitted with the separation ratios 9:7 or 3:1 expected for two complementary dominant genes [Χ^2^ = 0.461(< 3.84), Χ^2^ = 0.252(< 3.84), Χ^2^ = 0.667(< 3.84) and Χ^2^ = 0.378(< 3.84)] (Table 1).

**Table 1:** Responses of parents and progenies of experimental crosses of pepper lines ‘PBC932’ and ‘77013’ to infection by *Colletotrichum acutatum* at 7 days after inoculation. Separation ratios of disease-resistant and disease-susceptible plants in BC_4_S_1_, BC4S_2_–1, and BC_4_S_2_–2 populations fitted significantly with 9:7 or 1:3 Mendelian models

Parents or cross	Number of plants			Expected ratio (R:S) under independence	Χ^2^ (df = 1,*P* = 0.05 χ^2^ < 3.84)
	Total	R	S		
PBC932	6	6	0	1:0	
77013	6	0	6	1:0	
F_1_[77013 × PBC932]	6	6	0	1:0	
BC_4_S_1_	432	250	182	9:7	0.461
BC_4_S_2_–1	242	140	102	9:7	0.252
BC_4_S_2_–2	162	117	45	3: 1	0.667

**Fig. 2:**
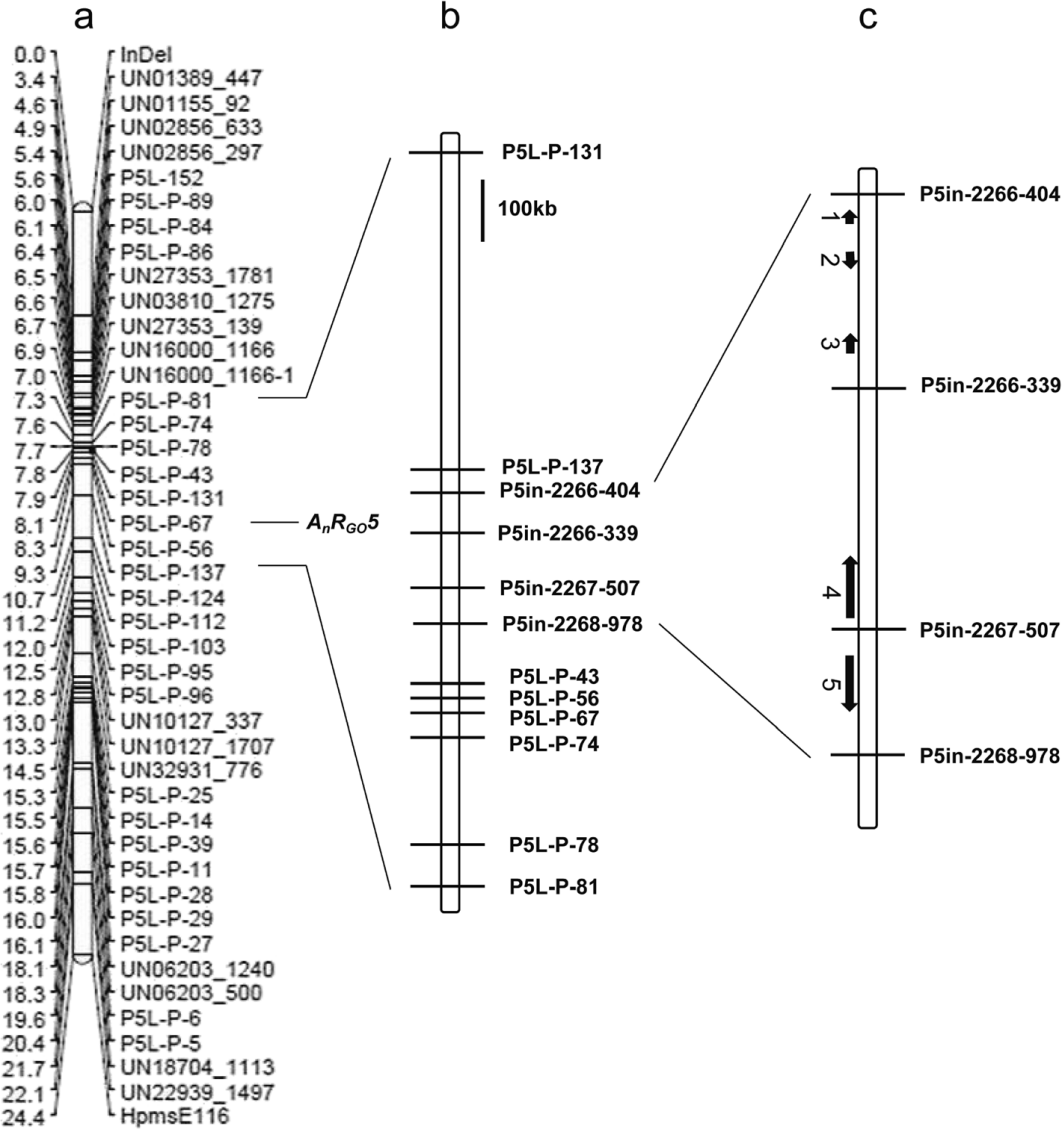
Genetic linkage mapping of preliminary position of *A*_*n*_*R*_*GO*_*5* on chromosome 5 of pepper based on BC_4_S_1_ [(‘77013’ × ‘PBC932’) × ‘77013’]. **a** Genetic linkage map constructed according to BC_4_S_1_ population containing 44 markers. **b** Confirmation of *A*_*n*_*R*_*Go*_*5* locus in interval between markers P5in-2266-404 and P5in-2267-978. **c** Predicted genes in *A*_*n*_*R*_*Go*_*5* region. Numbers indicate predicted genes (1: CA05g17700 2: CA05g17710 3: CA05g17720 4: CA05g17730 5: CA05g17740); arrows indicate direction of transcription

The genetic linkage map constructed according to the BC_4_S_1_ population contained a total of 44 markers, comprising 42 KASPar markers, 1 InDel marker, and 1 SSR marker. The full-length map was 24.4 cM long, and the average genetic distance between markers was 0.55 cM. By combining the map information with the true lesion diameter data, a QTL related to anthracnose resistance was predicted. The most closely linked QTL marker was P5L-P-67, with a contribution rate of 69.3% and an LOD of 24.37. A QTL with a 95% confidence interval, *AnR*_*GO*_*5,* was labeled between P5L-P-137 and UN16000_1166–1, and the genetic distance between these two markers was 2.33 cM. To narrow down the localization interval, R plants were selected from BC_4_S_1_ to construct the BC_4_S_2_–1 and BC_4_S_2_–2 populations. The individual plants in these populations were screened with nine markers between P5L-P-137 and UN16000_1166–1 to obtain the exchanged individual plants of BC_4_S_2_–44. The offspring of BC_4_S_2_–44 and the non-exchanged individual plant BC_4_S_2_–47 were planted separately, and the resistance phenotype of the green mature fruits was determined at 7 days after inoculation. The true lesion diameter values of the progeny of the two plants were significantly different. Thus, *A*_*n*_*R*_*GO*_*5* was located between the markers P5L-P-137 and P5L-P-81 (Fig. 2a). This interval contained 13 predicted genes.

To further narrow down the localization interval, the BC_4_S_3_ population containing 883 plants was constructed and analyzed. The physical locations of the *AnR*_*GO*_*5* gene cluster markers were found based on the genomic sequences of ‘CM334’ and ‘Zunla’. Four InDel markers were developed for this fine mapping interval on the basis of parental re-sequencing data, namely, P5in-2268-507, P5in-2268-339, P5in-2266-404, and P5in-2267-978. The exchanged individual plant BC_4_S_3_–6 was screened with 11 markers between P5L-P-137 and the labeled P5L-P-81. The offspring of BC_4_S_3_–6 and the non-exchanged individual plant BC_4_S_3_–417 were planted separately, and the phenotype of fruits was determined at 7 days after inoculation. The true lesion diameter values of the progeny of the two plants were significantly different. By comparing the genotypes and phenotypes of the exchanged plants BC_4_S_2_–44 and BC_4_S_3_–6, the major anti-anthracnose QTL *AnR*_*GO*_*5* in pepper at the green mature stage was localized between P5in-2266-404 and P5in-2267-978 (Fig. 2b, c). This interval contained five predicted genes.

Blastn searches for the five genes in this localization interval revealed that CA05g17700 encodes a pectin methylesterase-related protein; CA05g17710 and CA05g17720 encode unknown proteins; and CA05g17730 and CA05g17740 encode homologs of the ‘R1C-3-like’ potato-resistant late blight protein (Table 2). The CA05g17730 and CA05g17740 gene sequences were compared with sequences in the tomato genome database (SGN http://solgenomics.net). At the nucleotide level, CA05g17730 showed 85% similarity and CA05g17740 showed 88.1% similarity to Solyc05g043420.1.1 on chromosome 5 of tomato. Solyc05g043420.1.1 encodes a ‘nucleotide-binding site plus leucine-rich repeat’ (NBS-LRR) protein. This class of proteins is encoded by a large family of disease-resistance genes.

**Table 2:** Predicted genes in chromosome 5 region

ID	Predicted gene	Putative protein function
1	CA05g17700	Pectinesterase-3%2C putative
2	CA05g17710	Unknown protein
3	CA05g17720	Detected protein of unknown function
4	CA05g17730	Putative late blight resistance protein homolog R1C-3-like
5	CA05g17740	Putative late blight resistance protein homolog R1C-3-like

### Gene expression analysis

**Fig. 3:**
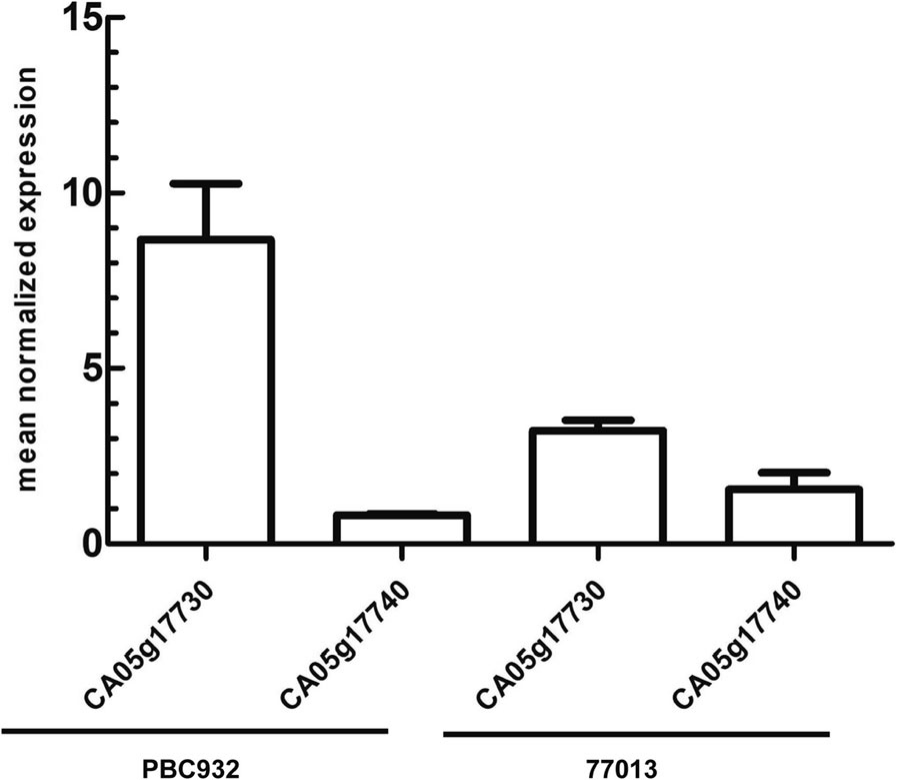
Results of RT-qPCR analyses to detect CA05g17730 and CA05g17740 gene transcript levels in pepper infected with *Colletotrichum acutatum* at 7 days after inoculation. Transcript level of CA05g17730, but not CA05g17740, differed significantly between ‘PBC932’ and ‘77013’

Specific fluorescent quantitative primers were used to analyze CA05g17730 and CA05g17740 transcript levels by RT-qPCR. There was no significant difference in the transcript level of CA05g17740 between ‘PBC932’ and ‘77013’, but the transcript level of CA05g17730 differed markedly between ‘PBC932’ and ‘77013’ (Fig. 3).

### Gene structural analysis

**Fig. 4:**
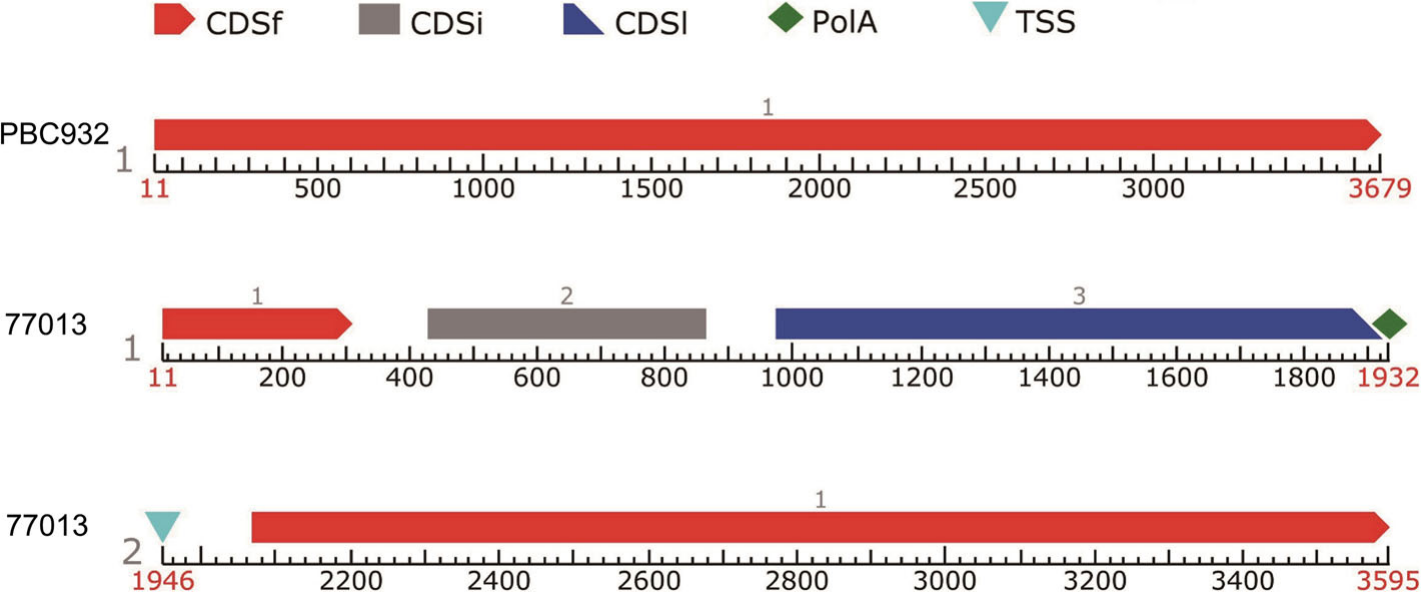
Gene structures of CA05g17730 in ‘PBC932’ and ‘77013’. One gene and one CDS of CA05g17730 was predicted in ‘PBC932’, and two genes and four CDSs were predicted in ‘77013’. CDSf:First (starting with start codon); CDSi: internal (internal exon); CDSl: last coding segment; PolyA: polyadenylic acid; TSS: position of transcription start (TATA-box position and score)

Primers were designed from the CA05g17730 gene sequence to amplify the full-length CA05g17730 gene sequence from ‘PBC932’ and ‘77013’. The full-length CA05g17730 was 3673-bp long in ‘PBC932’, and 3589-bp long in ‘77013’. Compared with the CA05g17730 sequence in‘PBC932’, that in ‘77013’ had a 19-bp deletion at 177 bp, an 8-bp deletion at 204 bp, a 15-bp deletion at 472 bp, a 42-bp deletion at 704 bp, and a 3-bp deletion at 3644 (Additional file 2: Figure S1). Prediction of the exon regions of CA05g17730 using tools at the Sol Genomics Network and the Softberry websites revealed that all deletions in the sequence in ‘77013’ were in exon regions. The missing parts of the CA05g17730 gene between ‘PBC932’ and ‘77013’ may result in functional differences in the CA05g17730 protein between these two lines. Using tools at the Softberry website to predict gene structure, one CDS in CA05g17730 was predicted in ‘PBC932’, and two genes and four CDSs were predicted in ‘77013’. The transcription of CA05g17730 in ‘77013’ was predicted to prematurely terminate at 1932 bp due to a base deletion, resulting in the loss of function of CA05g17730 in ‘77013’ (Fig. 4).

## Discussion

In this report, we fine mapped the locus resistant to *C*. *scovillei* from PBC932 in the 164Kb physical interval on P5 chromosome. This is more prices than that our lab once located the locus in the interval about 9.6 cM on the same chromosome and P7, P10 and P12 chromsomes [21]. Based a ‘PBC932’-derived map recently and a different resistance QTL to other *Colletotrichum* species was found on P2 as a different result [14]. Here linked to the locus, several markers (P5in-2266-404, P5in-2266-339, P5in-2267-507, P5in-2268-978) have been developed useful to molecular marker-assisted selection.

Gene CA05g17730 is one of the most suspect candidate resistant gene according our partial proves by now. In the 164Kb physical interval, only five genes were found including CA05g17730. Our analyses showed that the sequence of the CA05g17730 gene encoding homologes of Solyc05g043420.1.1, the ‘R1C-3-like’ putative late blight resistance protein and show different in the transcript level between resistant and recessive lines. Most R genes encodes the ‘nucleotide-binding site plus leucine-rich repeat’ (NBS-LRR) class of proteins, which contain a conserved nucleotide-binding (NB) site critical for ATP or GTP binding [26, 27]. Previous studies have explored the roles of NBS-LRR proteins in diverse plants including *Arabidopsis* [28], potato [29, 30], rice [31], corn [32], and tomato [33]. Further research is required to study CA05g17730 function and if it confers resistance in more detail.

In the PBC932 are other genes suspect involved the resistance to *C*. *scovillei*? In previous studies Lin et al. have considered two complementary dominant genes control resistance to *C. scovillei* in ‘PBC932’ [19]. Then Sun et al. confirmed, as Mahasuk et al. have reported [2, 16], the heredity of the PBC932 once have proved resistanceat green and red fruit stages are controlled by distinct genes withinthe same P5 genome interval with recombinant individuals [21]. Sun et al. have located several QTLs on P5, P7, P10, P12 chromsomes [21]. So there are more genes involved the resistance on different locus. Even on P5 chromsome, in the present study, the segregation ratios of resistance and susceptibility to *C. acutatum* in the BC4S_1_, BC4S_2_–1, and BC4S_2_–2 populations fitted significantly with a 1:3 or 9:7 Mendelian model, indicating that most of the genetic variation was explained by two complementary dominant genes. On fact, Gene CA05g17710 and CA05g17720 encode unknown function proteins.

## Conclusions

We narrowed down the major QTL resistant to *C. scovillei* interval to a 164 Kb region containing five genes. Homologe and transcript analyses indicated that CA05g17730 is the suspect candidate gene conferring resistance to *C. scovillei* at the mature green fruit stage of pepper. This finding provides new insights into the relationship between *C. scovillei* and host plant. These results will be useful for further research on the mechanisms of resistance to anthracnose, and for marker assisted selection for anthracnose-resistant capsicum lines.

## Methods

### Mapping population and pathogen

The female parent was the inbred line ‘77013’ (*C. annuum* L.), which was bred at the Institute of Vegetables and Flowers (IVF), Chinese Academy of Agricultural Sciences (CAAS) [21]. The paternal parent was ‘PBC932’ (*C. chinense* Jaqu.), which is resistant to *C. acutatum* and was provided by Dr. Wang Tiancheng, AVRDC [21]. The F_1_ generation was derived from the interspecific cross of *C. annuum* ‘77013’ × *C. chinense* ‘PBC932’. The F_1_ was backcrossed with ‘77013’ for several generations to obtain the BC_4_ population. Plants resistant to *C. acutatum* were selected from the BC_4_ population, and the BC_4_S_1_ population was obtained by selfing. Then, interspecific BC_1_S_1_, BC_1_S_2_, and BC_1_S_3_ progenies [(‘77013’ × ‘PBC932’) × ‘77013’] were obtained.

Using the single-spore isolation method [34], the isolate ‘Ca’, was collected and isolated from diseased fruit of pepper plants in Hunan province, China, in 2009. This isolate was positively identified as *C. acutatum* on the basis of its colony morphology and rDNA-ITS (ITS4/ITS5) sequence (Genbank accession No. KC936995) [21]. By blasting rDNA-ITS (ITS4/ITS5) sequence in NCBI, the isolate ‘Ca’ was reclassified as *C. scovillei*.

### Assessment of anthracnose resistance

Detached mature green fruits were inoculated by microinjection using the method developed at the AVRDC in 1999, with slight modifications. Mature green fruit were washed in distilled water and 75% ethanol. The *C. scovillei* isolate ‘Ca’ was cultured for 4–6 days in freshly prepared liquid Potato Dextrose Agar (PDA) medium with shaking at 100 rpm at 28 °C in the dark to promote conidia formation. Then, the liquid PDA medium was filtered through four layers of sterile gauze to obtain a conidial suspension. A 1-mm-deep wound was made by piercing the surface of mature green fruit, and 1 μl conidial suspension (concentration, 5 × 10^5^ ml·ml^− 1^) was injected into each wound site. Each fruit had two to six wounds depending on the fruit size. After inoculation, the fruit were placed in a plastic box with four layers of moist sterile filter paper to maintain humidity at about 95%. After incubation at 26 °C for 7 days, true lesion diameter (defined as the average lesion diameter (mm) over all lesions) was measured as an indicator of the resistance phenotype [35].

### Development of cluster-targeted molecular markers and linkage map

The *AnR*_*GO*_*5* gene cluster markers of one InDel marker and one SSR marker were screened in parental lines and the BC_1_ population [21, 36]. The physical map data for pepper lines ‘CM334’ and ‘Zunla’ [37, 38] were used for these analyses. The *AnR*_*GO*_*5* gene cluster markers were aligned to the ‘CM334’ genome sequence using the BlastN option online to determine the physical position (http://passport.pepper.snu.ac.kr/?t=PGENOME). Then, we developed markers in that physical interval. We developed KASPar markers based on the comparison of transcriptome sequences with the pepper genome information (http://peppergenome.snu.ac.kr/download.php) (Wang et al., unpublished data). From them, we selected 16 KASPar markers (Additional file 1: Table S1) on chromosome 5 around the position of *AnR*_*GO*_*5* reported previously [21]. Based on the re-sequencing data of ‘PBC932’ and ‘77013’, we searched for SNP loci in that physical interval, and 26 pairs of polymorphic KASPar primers were designed using Primer 5.0 software.

Total genomic DNA was extracted from the parental lines and the BC_4_S_1_, BC_4_S_2_, and BC_4_S_3_ populations using the CTAB method [39]. Markers were synthesized by the Sangon Biotechnology Co. (Beijing, China). In total, 42 markers were analyzed and their linkages determined using Join Map 4.0 software [40]. Map distances were calculated using the Kosambi function. The QTL analysis of the anthracnose disease scores was performed using MapQTL 6.0 with LOD 3.0 and a step size of 0.5 [41]. Major QTL were defined on the basis of the proportion of phenotypic variation explained (% E). To fine-map *AnR*_*GO*_*5*, the true lesion diameter values of homozygous recombinants and homozygous non-recombinants from each progeny family were compared using Student’s t test.

### Prediction of gene functions

The physical positions of *AnR*_*GO*_*5*-linked markers were determined according to a blastn search of the ‘CM334’ and ‘Zunla’ genomes. Information about the predicted genes in the region encompassing *AnR*_*GO*_*5* was collected by blastn searches of the Sol Genomics Network (SGN http://solgenomics.net) and The National Center for Biotechnology Information (NCBI http://www.ncbi.nlm.nih.gov) databases.

### RT-qPCR and candidate gene determination

The gene sequences were searched against the ‘CM334’ and ‘Zunla’ genome databases (http://passport.pepper.snu.ac.kr/?t = PGENOME;http://peppersequence.genomics.cn/page/species/index.jsp). Specific fluorescence quantitative primers (100–200 bp) were designed according to the coding sequence (CDS) of genes (Additional file 1: Table S2).

Healthy mature green fruits of ‘PBC932’ and ‘77013’ were selected and inoculated with the conidial suspension. The fruits were left attached to the plants. After 7 days, flesh near the lesions on fruits was collected and immediately frozen in liquid nitrogen. Total RNA was isolated using the SV Total Isolation System (Promega, Madison, WI, USA). First-strand cDNA was synthesized from RNA using 5X All-In-One RT MasterMix (Applied Biological Materials Inc., Richmond, Canada). The PCR analyses were conducted using gene-specific primers and GoTaq® qPCR Mix. The PCR analyses were conducted using a Light Cycler 480 II instrument (Roche Diagnostics Ltd., Rotkreuz, Switzerland). Each PCR mixture (20 μl volume) was subjected to the following thermal cycling conditions: 95 °C for 10 min; 40 cycles of 95 °C for 15 s, 55 °C for 30 s, and 72 °C for 30 s; and 95 °C for 15 s; 60 °C for 15 s; and 95 °C for 15 s. Three biological replications were included for each experiment. Relative expression levels were calculated using the 2^−∆∆Ct^ method [42]. Candidate genes were identified as those showing significant differences in transcript levels between ‘PBC932’ and ‘77013’ in RT-qPCR analyses.

### Gene sequence analyses and gene structure prediction

To obtain the whole sequence of candidate genes, each gene was amplified from the genome of ‘PBC932’ and ‘77013’ using gene-specific primer pairs (Additional file 1: Table S3). The products were sequenced by the Sangon Biotechnology Co. (Beijing, China), and sequences were assembled using SeqMan software. The sequences of each gene were compared between ‘PBC932’ and ‘77013’ using DNAMAN V6 software. The structures of the genes in the two parental lines were predicted using tools at the Softberry website (http://linux1.softberry.com/berry.phtml?topic=fgenesh&group=programs&subgroup=gfind).

## Supplementary information

**Additional file 1: Table S1.** Sequences of the SSR, InDel, and KASPar markers used in this study to map *AnR*_*GO*_*5* on chromosome 5. **Table S2.** Sequences of primers used to perform RT-qPCR experiments. **Table S3.** Sequences of primers used to clone candidate genes.

**Additional file 2: Figure S1.** Sequences of CA05g17730 gene in *Capsicum chinense* ‘PBC932’ and *Capsicum annuum* ‘77013’ obtained by PCR amplification and sequencing.

## Data Availability

The datasets used and/or analysed during the current study are available from the corresponding author on reasonable request.

## Abbreviations

O: The average lesion diameter (mm) over all lesions
R: Disease resistant
S: Disease susceptible
