# Supplementary material for: Fine mapping of the major anthracnose resistance QTL AnRGO5 in Capsicum chinense ‘PBC932’
Source: BMC Plant Biol. 2020 May 1;20:189. doi: 10.1186/s12870-019-2115-1 (PMC7195712; doi:10.1186/s12870-019-2115-1)
Supplement: Supplementary file 1 — Additional file 1: Table S1. Sequences of the SSR, InDel, and KASPar markers used in this study to map AnRGO5 on chromosome 5. Table S2. Sequences of primers used to perform RT-qPCR experiments. Table S3. Sequences of primers used to clone candidate genes. [file 12870_2019_2115_MOESM1_ESM.docx]

Table S1 Sequences of the SSR、Indel and Kaspar markers used in the present study to map the *AnR_GO_5* gene on chromosome 9.

| **Markers** | **Forward primer sequences 1 (5′–3′)** | **Forward primer sequences 2 (5′–3′)** | **Reverse primer sequences (5′–3′)** | **Marker Type** |
| --- | --- | --- | --- | --- |
| HpmsE116 | CATCTCTCCGTTGAATCTATTTCC |  | ACGGTCATCCATTAGAACCGTA | SSR |
| InDel | GGTATCTTATTTCATAGGGACCAGGCA |  | TTTGCGGTAGTGACAACAACTTTACAGCCA | Indel |
| P5in-2266-404 | ATGCATGAATGTGTCCAAGA |  | CATCATACCAAACACACCCT | Indel |
| P5in-2266-339 | TCACAAAGCTTCTTCTATTTTCT |  | TGATATGATAGAGTTTTGTGGC | Indel |
| P5in-2267-507 | AAGTGGTTCAAGCTTTGTTG |  | TCAAAGGAGAACAAGTACAGA | Indel |
| P5in-2268-978 | TGTATCACAAGACAACCAGGTAG |  | TTCAGTCTCACACACTAAGTTGT | Indel |
| P5L-P-103 | GAAGGTGACCAAGTTCATGCTATATACATCCCTCTAAGAATCCTAC | GAAGGTCGGAGTCAACGGATTATATACATCCCTCTAAGAATCCTAG | TGAAGATGAGAGGAACTACTTGAGAT | Kaspar |
| P5L-P-11 | GAAGGTGACCAAGTTCATGCTTTTCAAATTAGGGGTACATC | GAAGGTCGGAGTCAACGGATTTTTCAAATTAGGGGTACATT | AGAAGTCTGACCCAAACTTGTT | Kaspar |
| P5L-P-112 | GAAGGTGACCAAGTTCATGCTACCCACAGCTTTCATTGGG | GAAGGTCGGAGTCAACGGATTACCCACAGCTTTCATTGGA | TTGGCACTTTCAGGTCATAGTTTAG | Kaspar |
| P5L-P-124 | GAAGGTGACCAAGTTCATGCTGGATTTCGGGATCGACGT | GAAGGTCGGAGTCAACGGATTGGATTTCGGGATCGACGG | GGTAACGAACCTCTCGCCA | Kaspar |
| P5L-P-131 | GAAGGTGACCAAGTTCATGCTAGGAATATGTTGAACAAACGAC | GAAGGTCGGAGTCAACGGATTAGGAATATGTTGAACAAACGAT | AATACCTCAAGCTTGTCTTCTCCC | Kaspar |
| P5L-P-137 | GAAGGTGACCAAGTTCATGCTCGGCACATTTATCGAGGAGTT | GAAGGTCGGAGTCAACGGATTCGGCACATTTATCGAGGAGTC | GTGGGATCGAAAAATCTCAAAA | Kaspar |
| P5L-P-14 | GAAGGTGACCAAGTTCATGCTGATTCCGATGAAGGCAAAA | GAAGGTCGGAGTCAACGGATTGATTCCGATGAAGGCAAAT | TGAAAATGATGGAGGGAGAGC | Kaspar |
| P5L-P-152 | GAAGGTGACCAAGTTCATGCTTCCTATCTTCCCCCCATCTG | GAAGGTCGGAGTCAACGGATTTCCTATCTTCCCCCCATCTT | AGAAGCCTCAAGTGTCTTACGTGA | Kaspar |
| P5L-P-25 | GAAGGTGACCAAGTTCATGCTGAGGGTTGGTGACAAGATTGA | GAAGGTCGGAGTCAACGGATTGAGGGTTGGTGACAAGATTGG | GTTAGATCTCGTATCCCAATGCAGT | Kaspar |
| P5L-P-27 | GAAGGTGACCAAGTTCATGCTATACCCCTTAGGCGTTACG | GAAGGTCGGAGTCAACGGATTATACCCCTTAGGCGTTACA | CTAGATCTGCCCTTGGCAC | Kaspar |
| P5L-P-28 | GAAGGTGACCAAGTTCATGCTACCAGCGCCAATGGAGA | GAAGGTCGGAGTCAACGGATTACCAGCGCCAATGGAGC | ATCCGGTAACTATCAACTACGGTG | Kaspar |
| P5L-P-29 | GAAGGTGACCAAGTTCATGCTGAGGCATTACAGGGCAGGA | GAAGGTCGGAGTCAACGGATTGAGGCATTACAGGGCAGGG | AACTTATGAAGGTACACACACGCAT | Kaspar |
| P5L-P-39 | GAAGGTGACCAAGTTCATGCTCCCGACAAAGGTAAGCCAAC | GAAGGTCGGAGTCAACGGATTCCCGACAAAGGTAAGCCAAT | ACGAGTAACGTTTGGATGAAAGA | Kaspar |
| P5L-P-43 | GAAGGTGACCAAGTTCATGCTTTAAAGGATCACGTTTTTCCCT | GAAGGTCGGAGTCAACGGATTTTAAAGGATCACGTTTTTCCCC | GAACTTTCGATATTTTCAAGAAGGC | Kaspar |
| P5L-P-5 | GAAGGTGACCAAGTTCATGCTCTTACTCTCCACTTGAGATTTTCCG | GAAGGTCGGAGTCAACGGATTCTTACTCTCCACTTGAGATTTTCCA | ATGGTCATCGTCCAAGAAAGG | Kaspar |
| P5L-P-56 | GAAGGTGACCAAGTTCATGCTTTTTCTTGGAACGTGTTGGAGA | GAAGGTCGGAGTCAACGGATTTTTTCTTGGAACGTGTTGGAGC | AAGAACGAATACATCCTTGGAAAGT | Kaspar |
| P5L-P-6 | GAAGGTGACCAAGTTCATGCTCTAGTGTTGTTGGTATATCGTTGCC | GAAGGTCGGAGTCAACGGATTCTAGTGTTGTTGGTATATCGTTGCT | GAAATAACCGGGCTAAAAGAACC | Kaspar |
| P5L-P-67 | GAAGGTGACCAAGTTCATGCTGCAACATGGGATGAGTTAACAAG | GAAGGTCGGAGTCAACGGATTGCAACATGGGATGAGTTAACAAC | TTCAGATGGGATGGTATATAATGGT | Kaspar |
| P5L-P-74 | GAAGGTGACCAAGTTCATGCTCCGTGCTCGAGGAATTAGC | GAAGGTCGGAGTCAACGGATTCCGTGCTCGAGGAATTAGA | TATAAGCTCATGACAACCTAGTATG | Kaspar |
| P5L-P-78 | GAAGGTGACCAAGTTCATGCTAAAATCTTCAGATGCACCGG | GAAGGTCGGAGTCAACGGATTAAAATCTTCAGATGCACCGA | AACCTTATGGCGGCG | Kaspar |
| P5L-P-81 | GAAGGTGACCAAGTTCATGCTATTGAGCTTCAGGACAACAAGTTTA | GAAGGTCGGAGTCAACGGATTATTGAGCTTCAGGACAACAAGTTTG | CGGAAACGAAGAAGCTCTTAGA | Kaspar |
| P5L-P-84 | GAAGGTGACCAAGTTCATGCTATGGGTATAAAGAAAGC | GAAGGTCGGAGTCAACGGATTATGGGTATAAAGAAAGT | CCAAAACCAAGATATTTGAGGATC | Kaspar |
| P5L-P-86 | GAAGGTGACCAAGTTCATGCTAATCTGGCCCTAGCAAATTATTCT | GAAGGTCGGAGTCAACGGATTAATCTGGCCCTAGCAAATTATTCG | AGCATGTGCTAAGGGAGATGG | Kaspar |
| P5L-P-89 | GAAGGTGACCAAGTTCATGCTACTGGCAACATCATCTACATGC | GAAGGTCGGAGTCAACGGATTACTGGCAACATCATCTACATGA | CTCGCTTACCCCATTCGT | Kaspar |
| P5L-P-95 | GAAGGTGACCAAGTTCATGCTAACCAGGGGTCGTTGACA | GAAGGTCGGAGTCAACGGATTAACCAGGGGTCGTTGACG | CCATCTGGCTTGTCACCACTAG | Kaspar |
| P5L-P-96 | GAAGGTGACCAAGTTCATGCTGTTGTCCGGTTAGTGGGATTAG | GAAGGTCGGAGTCAACGGATTGTTGTCCGGTTAGTGGGATTAT | ACGCCATGTCAAGAAGCAAA | Kaspar |
| UN32931_776 | GAAGGTGACCAAGTTCATGCTACGATATATGTTCAAGAAGATGTTTTCGAACG | GAAGGTCGGAGTCAACGGATTTTGTTTTCCCCTACATCAACAGCCT | TTGTTTTCCCCTACATCAACAGCCT | Kaspar |
| UN01155_92 | GAAGGTGACCAAGTTCATGCTCAATTGCTAGCATTGCTTCAATTCTTTTAGTA | GAAGGTCGGAGTCAACGGATTTTGCTAGCATTGCTTCAATTCTTTTAGTG | TCCTCTTGCTGGTAAATGTSATGGC | Kaspar |
| UN01389_447 | GAAGGTGACCAAGTTCATGCTCGGGGAATTCTACAGTAAAGTGAATGTATAA | GAAGGTCGGAGTCAACGGATTCGGGGAATTCTACAGTAAAGTGAATGTATAG | TGAAGGAATGCCTATGTACGGAAGG | Kaspar |
| UN02856_297 | GAAGGTGACCAAGTTCATGCTGACCAGTCCAACCACCTCCACCG | GAAGGTCGGAGTCAACGGATTGACCAGTCCAACCACCTCCACCA | GCCCGGGCCCATATCCATACGAATA | Kaspar |
| UN02856_633 | GAAGGTGACCAAGTTCATGCTAGAGAAGCCCAAAGAGCCCGAAAAA | GAAGGTCGGAGTCAACGGATTAGAGAAGCCCAAAGAGCCCGAAAAG | TTTRGGCTTTGGAGGCTCCTTGGGC | Kaspar |
| UN03810_1275 | GAAGGTGACCAAGTTCATGCTTTCCTAACAAAGGCGACTCGATATATATTTA | GAAGGTCGGAGTCAACGGATTCCTAACAAAGGCGACTCGATATATATTTG | CAGTTGCAGAAACAAAGAAAGGTGG | Kaspar |
| UN06203_1240 | GAAGGTGACCAAGTTCATGCTATGGGACGTTGTATCAAACGAGACA | GAAGGTCGGAGTCAACGGATTATGGGACGTTGTATCAAACGAGACT | AGCTCGCTGGTGATGATRGTCGWCT | Kaspar |
| UN06203_500 | GAAGGTGACCAAGTTCATGCTTGTGTGTGGAAGAAGAAGAGATATGGAAGATA | GAAGGTCGGAGTCAACGGATTTGTGTGGAAGAAGAAGAGATATGGAAGATG | TCCTTGAGGTTTCACTATTCTCCTTGGT | Kaspar |
| UN10127_1707 | GAAGGTGACCAAGTTCATGCTAAAGAACTTGGCAGCAGTCCATTCG | GAAGGTCGGAGTCAACGGATTAAAGAACTTGGCAGCAGTCCATTCA | ACCTTACGTATTTGTACAAGCTGCTTTCT | Kaspar |
| UN10127_337 | GAAGGTGACCAAGTTCATGCTACAACATTATCAAAAGTGATGGAATGTGGTTC | GAAGGTCGGAGTCAACGGATTACAACATTATCAAAAGTGATGGAATGTGGTTT | TGAAACTGAGGGTCGGGACAAGAA | Kaspar |
| UN16000_1166 | GAAGGTGACCAAGTTCATGCTTCAAYAACTGGTGTTGCAATTCCCT | GAAGGTCGGAGTCAACGGATTTCAAYAACTGGTGTTGCAATTCCCC | TGGTTGATAGCAGCTATGGATTCTGAG | Kaspar |
| UN16000_1166-1 | GAAGGTGACCAAGTTCATGCTAGCTATGGATTCTGAGGAGGGTAAAAA | GAAGGTCGGAGTCAACGGATTAGCTATGGATTCTGAGGAGGGTAAAAG | TGCTCATGTTCAACCTCTGAATCAGA | Kaspar |
| UN18704_1113 | GAAGGTGACCAAGTTCATGCTTTTGGCAGTTCACACATGTCACGTAA | GAAGGTCGGAGTCAACGGATTTTTGGCAGTTCACACATGTCACGTAT | GCACCTACTATCAGATGCAAACGCG | Kaspar |
| UN22939_1497 | GAAGGTGACCAAGTTCATGCTTAGCATGAAACTGGTTATGCCCACTAAGA | GAAGGTCGGAGTCAACGGATTTAGCATGAAACTGGTTATGCCCACTAAGG | YGGATGACTTCTGGAGAACATTTAGCATTCA | Kaspar |
| UN27353_139 | GAAGGTGACCAAGTTCATGCTCCTCTGAGACATTGTGACTAATTTTAGCGCG | GAAGGTCGGAGTCAACGGATTAGCTAAGATGCTGCACAACTGTTCCA | AGCTAAGATGCTGCACAACTGTTCCA | Kaspar |
| UN27353_1781 | GAAGGTGACCAAGTTCATGCTGCTCGGTTCTTCCATCCAAGATAAAGCGT | GAAGGTCGGAGTCAACGGATTTCGGTTCTTCCATCCAAGATAAAGCGC | GGCGTGTTGAAAGACTTCAGCCCAA | Kaspar |

Table S2 Sequences of the primers used to perform RT-qPCR experiments

| Gene ID | Primers | chromosome | Position on chromosome | Amplicon length(bp) | Forward primer sequrnce | Reverse primer sequence |
| --- | --- | --- | --- | --- | --- | --- |
|  |  |  |  |  |  |  |
| CA05g17730 | 7305 | Chr5 | 226734380 | 156 | AAGATGTGATTTTGGAGGGC | ATTCGAGATTGTGGAAGGTGA |
| CA05g17740 | 7405 | Chr5 | 226767303 | 171 | AGGTGGTAGCGAACAGAGTTG | GAATAAATGCCCTCATCAAAAC |

Table S3 Sequences of the primers used to clone the candidate genes.

| **Markers** | **Forward primer sequences (5′–3′)** | **Reverse primer sequences (5′–3′)** | **Product size** |
| --- | --- | --- | --- |
| 730P-23 | TGCGAATCTCCATTATCTACCC | ACAGGAAATCTTGAGACGCTCT | 2600 |
| 730P-35 | ATTGTAGGATTTGAGGAGGAGA | TCTAACTTGGATAGAAGGCACA | 3231 |
| 730P-36 | CATGGAGTGAGTGGGTCAGTAA | GACGCTCTAATGTTGTTAGATT | 3675 |
| 730P-36-1 | TTGGAGCACTTATCAGGGAGGT | AAATTCAGATGGAATGGCGTAC | 988 |
| 730P-36-4 | CCACCTGAATATGTAGCGTGAT | CTTTCTTCTCCGAATACCCTTT | 2487 |
| 730P-36-5 | TTTAGCAGCTATCATCTGTTGT | GATTATCCACTCTATCTCCTCC | 1717 |
