# Supplementary figures and images for: Fine mapping of the major anthracnose resistance QTL AnRGO5 in Capsicum chinense ‘PBC932’
Source: BMC Plant Biol. 2020 May 1;20:189. doi: 10.1186/s12870-019-2115-1 (PMC7195712; doi:10.1186/s12870-019-2115-1)

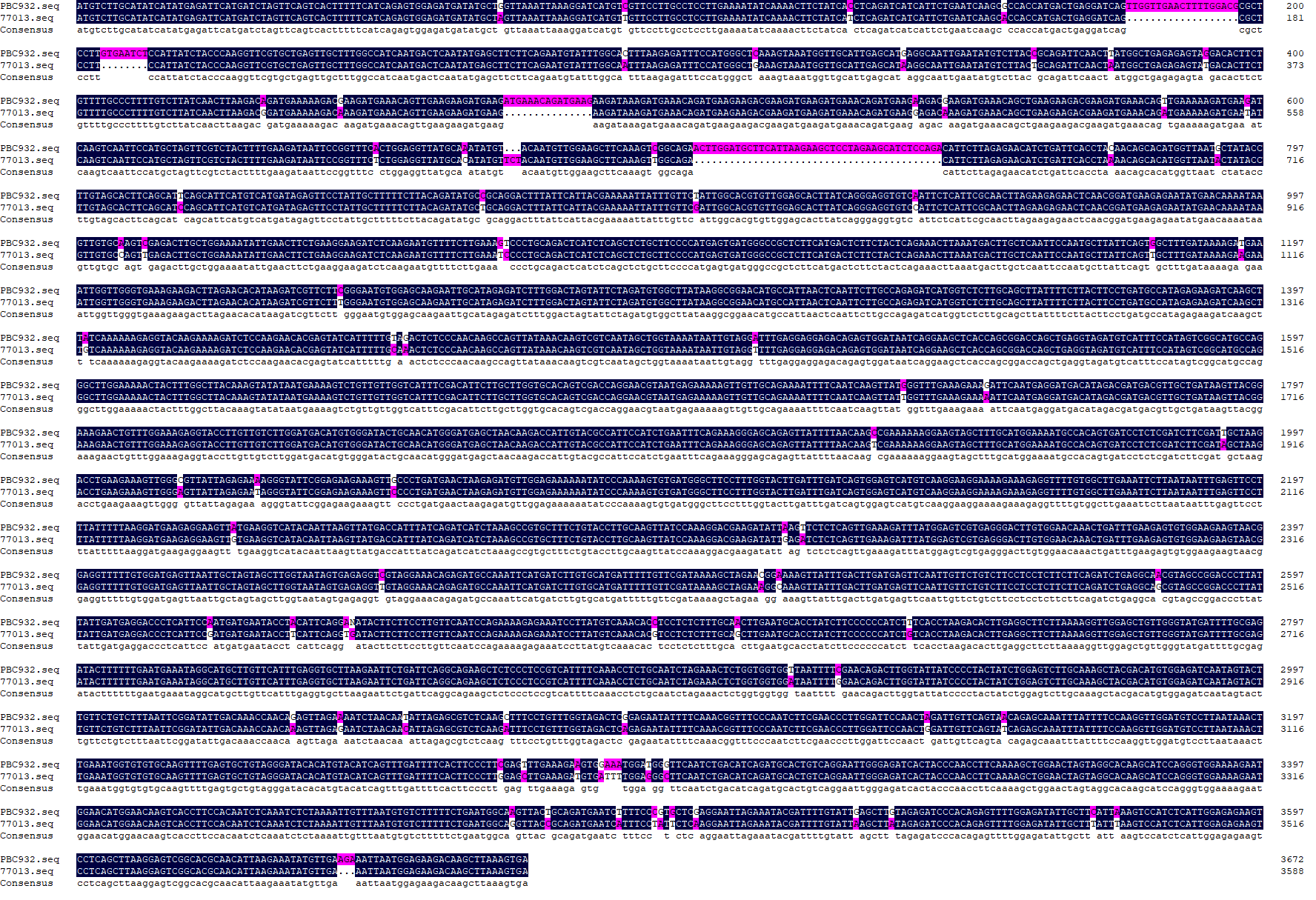

Supplement: Supplementary file 2 — Additional file 2: Figure S1. Sequences of CA05g17730 gene in Capsicum chinense ‘PBC932’ and Capsicum annuum ‘77013’ obtained by PCR amplification and sequencing. [file 12870_2019_2115_MOESM2_ESM.tif]
